# The effect of intrinsic image memorability on recollection and familiarity

**DOI:** 10.3758/s13421-020-01105-6

**Published:** 2020-11-23

**Authors:** N. Broers, N.A. Busch

**Affiliations:** 1 Institute of Psychology and Sports Science, University of Münster, Münster, Germany; 2 Otto Creutzfeld Center for Cognitive and Behavioral NeuroscienceUniversity of Münster, Münster, Germany

**Keywords:** Memorability, Recollection, Familiarity, Natural scenes, Recognition

## Abstract

Many photographs of real-life scenes are very consistently remembered or forgotten by most people, making these images intrinsically memorable or forgettable. Although machine vision algorithms can predict a given image’s memorability very well, nothing is known about the subjective quality of these memories: are memorable images recognized based on strong feelings of familiarity or on recollection of episodic details? We tested people’s recognition memory for memorable and forgettable scenes selected from image memorability databases, which contain memorability scores for each image, based on large-scale recognition memory experiments. Specifically, we tested the effect of intrinsic memorability on recollection and familiarity using cognitive computational models based on receiver operating characteristics (ROCs; Experiment 1 and 2) and on remember/know (R/K) judgments (Experiment 2). The ROC data of Experiment 2 indicated that image memorability boosted memory strength, but did not find a specific effect on recollection or familiarity. By contrast, ROC data from Experiment 2, which was designed to facilitate encoding and, in turn, recollection, found evidence for a specific effect of image memorability on recollection. Moreover, R/K judgments showed that, on average, memorability boosts recollection rather than familiarity. However, we also found a large degree of variability in these judgments across individual images: some images actually achieved high recognition rates by exclusively boosting familiarity rather than recollection. Together, these results show that current machine vision algorithms that can predict an image’s intrinsic memorability in terms of hit rates fall short of describing the subjective quality of human memories.

Our visual memory capacity for real-life scenes and objects is one of the most impressive feats of human cognition (Brady, Konkle, Alvarez, & Oliva, 2008; Standing, 1973). While memories of specific images are in part influenced by individual factors such as interest (Hidi, 1990) or expertise (Curby, Glazek, & Gauthier, 2009), it has been shown that many images are in fact consistently remembered or forgotten across many observers (Isola, Xiao, Parikh, Torralba, & Oliva, 2014; Bylinskii, Isola, Bainbridge, Torralba, & Oliva, 2015; Bainbridge, Isola, & Oliva, 2013; Bainbridge, 2020). This consistency of an image’s memorability spans a wide array of different picture presentation times (Mancas & Le Meur, 2013; Broers, Potter, & Nieuwenstein, 2018; Goetschalckx, Moors, Vanmarcke, & Wagemans, 2019b; Mohsenzadeh, Mullin, Oliva, & Pantazis, 2019), study and test intervals (Goetschalckx, Moors, & Wagemans, 2018; Isola et al., 2014) and experimental paradigms (Bylinskii et al., 2015; Bainbridge, 2020; 2017; Jaegle et al., 2019), implying that memorability is largely independent of personal or situational factors (Bainbridge, 2019). While some images contain information one would expect to be highly memorable (e.g., close-ups of humans/animals, distinctive objects that appear out of context), many memorable images are not particularly conspicuous and observers cannot accurately judge whether an image is memorable or not (Isola et al., 2014) (see Fig. 1 for example images). Most previous studies have focused on the application of machine vision algorithms to predict memorability as accurately as possible and to identify the image information that makes an image memorable (Isola et al., 2014; Bylinskii et al., 2015; Khosla, Raju, Torralba, & Oliva, 2015; Goetschalckx, Andonian, Oliva, & Isola, 2019a). Convolutional neural networks (CNNs) have been particularly successful at predicting image memorability (Khosla et al., 2015). These networks are composed of multiple processing layers that learn representations of input data with increasing levels of abstraction, setting new benchmark performances in scene and object recognition (LeCun, Bengio, & Hinton, 2015; Simonyan & Zisserman, 2015). Importantly, these studies have quantified memorability by assessing hit rates in image recognition tasks. However, the cognitive processes underlying these recognition decisions are largely unknown (but see: Akagunduz et al., 2019.

**Fig. 1:**
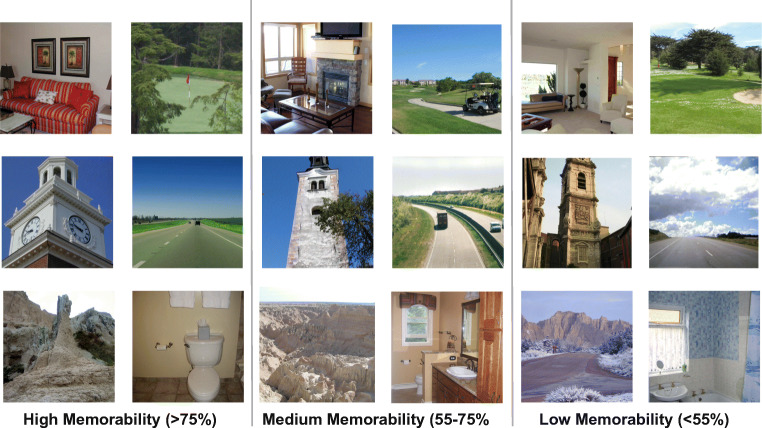
Pictures across the Memorability spectrum. Exemplars from six semantic categories (bedroom, golf course, tower, highway, badlands and bathroom) are shown for highly, medium and low memorable images, as quantified by Bylinskii, Isola, Bainbridge, Torralba, and Oliva (2015). While some highly memorable exemplars appear to be more distinct compared to their conceptual counterparts, considerable differences are not obvious to the naked eye. Of course, some images have special or peculiar content that evokes the reaction “it must be memorable” but people are generally incapable of judging whether an image is memorable or not (Isola et al., 2014)

It has long been acknowledged that old items can be recognized based on a feeling of familiarity or recollection of specific contextual details about the study event (Mandler, 1980; Yonelinas, 2001). The famous “butcher-on-the-bus” anecdote by Mandler (1980) perfectly exemplifies these two phenomenologies during recognition. The anecdote concerns an encounter with a man on a bus whose familiar face prompts a query in memory. The observer might not be able to retrieve additional information about the man, despite being confident of knowing him. Thus the man only feels familiar. If a query in memory yields additional information about the man, the observer would then recollect that he is in fact the butcher from the local supermarket. Two of the most prominent methods for assessing recollection and familiarity are Remember/Know (R/K) statements (Tulving, 1985) and receiver operating characteristics (ROCs; Yonelinas and Parks, 2007). In R/K tasks, participants indicate directly, after an old/new statement, whether they remember specific episodic details about the item (recollection) or whether they only know that the item is old (familiarity) (Tulving, 1985; Gardiner, Ramponi, & Richardson-Klavehn, 2002).

ROCs on the other hand are an indirect tool to index recollection and familiarity (Yonelinas & Parks, 2007). An ROC is a function that relates the hit rate to the false-alarm rate across different levels of an increasingly relaxed response criterion, such as decision confidence (see Fig. 2 for illustrations). ROCs have been explored with different computational models that make different assumptions on the cognitive mechanisms underlying recognition. According to dual-process signal detection (DPSD) models, the shapes of ROC curves can reflect two distinct memory processes (Yonelinas & Parks, 2007). First, recollection is treated as an all-or-none process, where information about an item is only recollected if its memory strength exceeds a certain threshold. Recollection-associated responses are assumed to be more confident on average for hits than for false alarms, resulting in a “hockey-stick”-shaped ROC. Thus, the intercept is an index of recollection and bent upwards for most conservative responses in z-transformed ROC shapes (see Fig. 2a). Secondly, familiarity is treated as a signal-detection process, where an item is accepted as old if its memory strength exceeds a decision criterion. Familiarity-associated responses produce curvilinear ROCs, where the area between the curve and the chance diagonal is an index of familiarity, and linear z-transformed ROCs, where the intercept is an index of recognition accuracy (see Fig. 2b). Importantly, according to DPSD models, the difference between recollection and familiarity is conceptually distinct from differences in decision confidence, although they may be correlated empirically. Successful recognition always depends on both processes, but if recollection fails, recognition is assumed to rely on familiarity (Yonelinas, Aly, Wang, & Koen, 2010). Thus, the two processes are assumed to be parallel, but functionally and neuroanatomically distinct (Eichenbaum, Yonelinas, & Ranganath, 2007). By contrast, single-process signal detection models assume that recollection and familiarity are both simply a measure of memory strength, with recollection reflecting higher memory strength than mere familiarity (Donaldson, 1996; Wixted & Stretch, 2004). A particularly successful variant of single-process models is the Unequal Variance Signal Detection (UVSD) Model, which assumes that the distribution of old items has greater variance than the distribution of new items. It is important to emphasize that neither model denies that recollection and familiarity are phenomenologically distinct ways of remembering, whether or not they may reflect distinct cognitive processes.

**Fig. 2:**
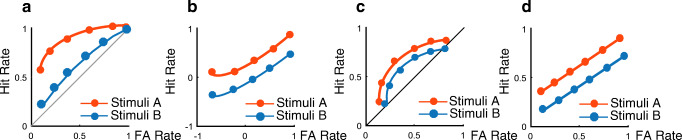
ROC curves and models of recognition memory. ROCs are functions relating the proportion of correctly recognized old items to the proportion of falsely recognized new items across different levels of a response criterion, typically measured as decision confidence (1 = “Sure New” to 6 = “Sure Old”). The function is cumulative and represents, from left to right, an increasingly relaxed criterion. The leftmost point on the curve represents the hit rate and false-alarm rate at the most conservative response criterion (6 = “sure old”), the next point represents hit rate and false-alarm rate of the two most conservative criteria (6 = “sure old” and 5 = “probably old”), etc. The area under the curve represents recognition performance, ranging from 1 (perfect accuracy) to 0.5 (guessing, i.e., a ROC falling on the diagonal). To compare ROC curves statistically, hit rates and false-alarm rates are typically standardized and plotted in z-space (Fig. 2b and d). In Fig. 2a and b, Stimulus A is associated with greater recollection and familiarity: the ROC curve is asymmetric and is thus bent upwards for most conservative responses, whereas area under the curve towards the chance diagonal is an index for increased familiarity. In z-space (Fig. 2b), this asymmetry leads to a slope less than 1. In Fig. 2c and d, ROCs and z-ROCs are shown for two stimuli recognized only by familiarity. ROC curves for both stimuli are curvilinear with larger area under the curve for stimulus A whereas in Fig. 2d, z-ROCs are linear. A larger z-intercept reflects greater memory strength

Interestingly, the effect of experimental manipulations on recollection and familiarity is quite variable (see Yonelinas, 2002 for a comprehensive review). For example, deep encoding compared to shallow encoding improves recollection more than it improves familiarity (Gardiner, 1988). In a similar vein, full attention conditions compared to diverted attention conditions are more associated with recollection rather than with familiarity (Yonelinas, 2001). However, other factors such as item repetition affect recollection and familiarity to a similar extent (Gardiner, Kaminska, Dixon, & Java, 1996). Processing fluency (i.e., how easily an item is processed, Rajaram, 1993) and rote rehearsal (Dobbins, Kroll, & Yonelinas, 2004) even influence familiarity more than recollection. Consequently, to which degree scenes across the memorability spectrum produce different kinds of memories is an open question yet to be resolved.

In the present study, we investigated whether intrinsic image memorability is associated with recollection and familiarity to a similar or different extent, using ROC curves (experiments 1 and 2) and R/K judgments (Experiment 2). Moreover, we investigated how the nature of memorability can be accounted for by cognitive computational models. While neural networks can predict how well people will recognize a scene based on a statistical analysis of image content (e.g., Khosla et al.,, 2015), it is unclear which kinds of memory representations support these recognition decisions. Importantly, different types of memory representations associated with different memory experiences activate different neural structures in the medial temporal lobe (Eichenbaum et al., 2007, e.g.,; Kafkas & Montaldi, 2012) and are associated with distinct event-related potentials in the EEG (Tsivilis, Otten, & Rugg, 2001; Rugg & Curran, 2007). Thus, any theory of memorability has to take the phenomenology of remembering into account. To this end, we compared how well recognition ROC curves are fitted by DPSD and UVSD models, and how their model parameters differ between highly and low memorable images.

## Experiment 1

### Methods

#### Participants

Fifty participants (31 female, mean age = 29.06) were recruited from the University of Muenster, Essen University Hospital, Open University Hagen and the University of Duisburg/Essen. All participants provided written informed consent. Participation was compensated with course credit (for students) or was voluntary. Four participants were excluded from analysis due to incomplete data sets. Another participant was excluded due to an unusual shape of the ROC curve, which could not be fit with any model. The study was approved by the ethics committee of the faculty of psychology and sports science, University of Muenster.

#### Apparatus and materials

Stimulus presentation and response logging was controlled with *PsychoPy v1.83.04* experimental software (Peirce, 2007), running on a Toshiba Satellite with 2.53 GHz Intel Core processor, 8 GB RAM and a Windows 7 64-bit operating system. Stimuli were presented on a 19-inch CRT monitor, with a 1280x768 resolution and a 60-Hz refresh rate.

Our stimulus set was comprised of 660 images. We extracted 355 pictures from the memorability image database FIGRIM (Bylinskii et al., 2015) and 305 images from the database established by Isola, Xiao, Parikh, Torralba, and Oliva (2014). A total of 241 different semantic categories were depicted in the images (see Table 4 in the Appendix for a distribution of unique semantic categories per condition). Each memorability category comprised an equal number of images, evenly split between the indoor/outdoor scene category.

The images from the *FIGRIM* database were shrunk to a resolution of 250x250 *px*, the same size as that of the pictures from Isola et al., (2014). Previous research has shown that memorability remains robust against overall decreases in picture size (Goetschalckx et al., 2019b). In order to avoid a confound of memorability and specific image content, this selection included only images without added elements such as text objects, and no close-up shots of human or animal faces. Since faces contribute to an image’s memorability (Isola et al., 2014; Khosla et al., 2015), we thereby excluded a number of images that were found highly memorable in previous studies. Images were categorized according to the memorability scores provided by Isola et al., (2014) and Bylinskii et al., (2015), which represent hit rates in online recognition memory experiments obtained from large samples of participants. Memorability scores > 75% were categorized as high memorability (hi-mem), scores < 75% and > 55% were categorized as intermediate memorability (mid-mem), and scores < 55% were categorized as low memorability (low-mem). Each category comprised 220 images with equal numbers of indoor and outdoor scenes. Each image was a target picture for one half of all participants and a foil picture for the other half. Memorability category and indoor/outdoor category were counterbalanced between the two sets of images. Mean scores per Memorability category and indoor/outdoor scene gist can be seen in Table 1.

**Table 1:** Mean memorability scores and mean hit-rates and false-alarm rates per memorability category and indoor/outdoor scene gist in Experiment 1

Memorability	Scene gist	Memorability score	Hit-rate Exp 1	False-alarm rate Exp 1
High	Indoor	0.87	0.65	0.32
High	Outdoor	0.86	0.64	0.30
Medium	Indoor	0.66	0.56	0.35
Medium	Outdoor	0.67	0.57	0.39
Low	Indoor	0.45	0.49	0.37
Low	Outdoor	0.49	0.54	0.36

#### Procedure

**Fig. 3:**
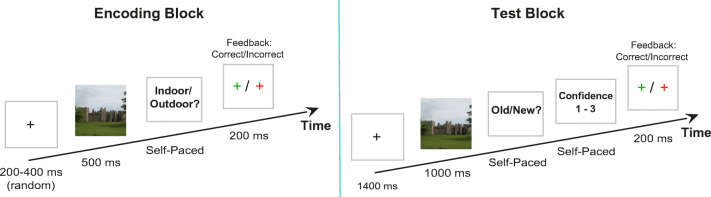
Illustration of a trial sequence. Encoding Block: Each trial started with a fixation cross for 200–400 ms, followed by a picture presented for 500 ms, a decision prompt (indoor versus outdoor scene) and feedback. Test block: Each trial started with a fixation cross presented for 1400 ms, followed by a picture presented for 1000 ms. Participants had to indicate with a button press whether the picture was old or new and how confident they were about their decision on a scale from 1 (sure old/new) to 3 (unsure old/new)

Image memory was tested in a recognition task with separate encoding and test blocks, separated by a 10-min break (Fig. 3).

In the encoding block, participants were instructed to memorize all images (330 in total, 110 per memorability category) while simultaneously categorizing each image as indoor or outdoor as fast as possible by pressing one of two response buttons. Trials started with a fixation cross (200 to 400-ms duration), followed by a scene image (500-ms duration), followed by a response prompt (indoor vs. outdoor). To keep participants engaged with the task, accuracy feedback was provided after each response by briefly turning the fixation cross red (error) or green (correct).

In the test block, participants were instructed to categorize each image as old or new, and to rate their confidence in their decision on a three-point scale, with no emphasis on response speed. All images from the encoding block were presented intermixed with 330 new foil images. Trials started with a fixation cross, followed by a scene image (1000-ms duration), followed by response prompts for the old/new and confidence reports. After the two reports were given, feedback about the old/new decision was provided. 1 Note that this paradigm with separate phases for encoding and test diverges from most previous studies of image memorability, which used a continuous recognition task (e.g., Isola et al., 2014; Bylinskii et al., 2015) where encoding and testing happen simultaneously.

#### Analysis

Performance was quantified separately for each individual image by calculating hit rates, false-alarm rates, and *d’* (Green and Swets, 1966). These performance indices were obtained by collapsing data across all participants. Hit rates and false-alarm rates were adjusted to avoid extreme values of 1 and 0, respectively, by adding 0.5 to both the number of hits and the number of false alarms, and adding 1 to both the number of old and new items, before calculating the hit and false-alarm rates (Snodgrass & Corwin, 1988; Hautus, 1995).

Moreover, hit rates, false-alarm rates, and *d’* were quantified separately for each participant and the three memorability categories by collapsing data across all images within a category. In addition, we analyzed each participant’s ROC curve by computing the area under each curve (AUC) using the trapezoidal rule for numerical integration (Wickens, 2002), which does not require a theoretical model of the ROCs. Performance measures were compared between memorability categories using paired, two-tailed *t* tests. Effect sizes of these analyses are reported as Cohen’s *d* (Cohen, 1988), computed according to Lakens (2013).

Finally, ROC curves were fitted with a DPSD model (Yonelinas, 1994) and a UVSD model (Mickes, Wixted, & Wais, 2007) using ROC Toolbox for MATLAB by Koen, Barrett, Harlow, and Yonelinas (2017). The UVSD model assumes that the distributions of memory strength of old items and new items overlap to a certain extent. The model parameter *d’*, or sensitivity, is an index of this overlap with larger values indicating less overlap, and thus better recognition performance. The second parameter (*Vo*) is an index of the variability of the old item distribution, with the assumption that memory strength of old items may be more variable than the strength of new items. In the DPSD model, the recollection parameter (*Ro*) represents the probability that participants recollect at least some aspect of the study event, whereas familiarity is represented by *d’*, with larger sensitivity indicating greater familiarity.

We first considered whether the models generally provide a statistically acceptable account of the individual participant data based on the *G*-test of goodness-of-fit (Koen, Aly, Wang, & Yonelinas, 2013). The test estimates the discrepancy between the expected values and the actual observed values in the model. If the test yields a value smaller than the 5% significance level, it is concluded that the given model deviates significantly from the data and is thus rejected (McDonald, 2009). We then compared performance between models on the basis of the Bayesian Information Criterion (BIC). The aim of the BIC is to obtain the posterior probability of the model given the data. The smaller the BIC for one model versus the other, the larger the posterior probability given the data (Schwarz & et al. 1978; Lewandowsky & Farrell, 2010). Both indices were applied to the aggregate as well as individual participant data. The model with lower BICs in 80% of participants was declared the winning model, on the condition that it has a statistically acceptable account of the data in more than 80% of participants, based on the *G*-statistic. Given that the parameters of the UVSD model allow for greater flexibility, the UVSD model has an a priori advantage at fitting a wider range of ROC data (Klauer & Kellen, 2011). Therefore, we complemented the comparison of fit statistics by testing which parameters of which model were most strongly associated with memorability. Importantly, a model with a superior model fit due to overfitting could potentially turn out to show only weak association with memorability.

### Results experiment 1

#### Replication of memorability

Across images, memorability scores obtained in previous studies (Bylinskii et al., 2015) were positively correlated with the hit rates (*r* = 0.34, *p* < 0.001, *d* = 0.73) and negatively correlated with false-alarm rates (*r* = − 0.17, *p* < 0.001, *d* = − 0.34) obtained for the same images in the present study. This resulted in a strong correlation between recognition sensitivity *d’* and memorability scores (Spearman’s *ρ* = 0.41, *p* < 0.001, *d* = 0.91). In spite of this consistency with previous studies, hit rates in the present study were overall consistently lower than hit rates/memorability scores obtained for the same images by Bylinskii et al., (2015, *t*(659.00) = 12.77, *p* < 0.001, *d* = 0.50).

**Fig. 4:**
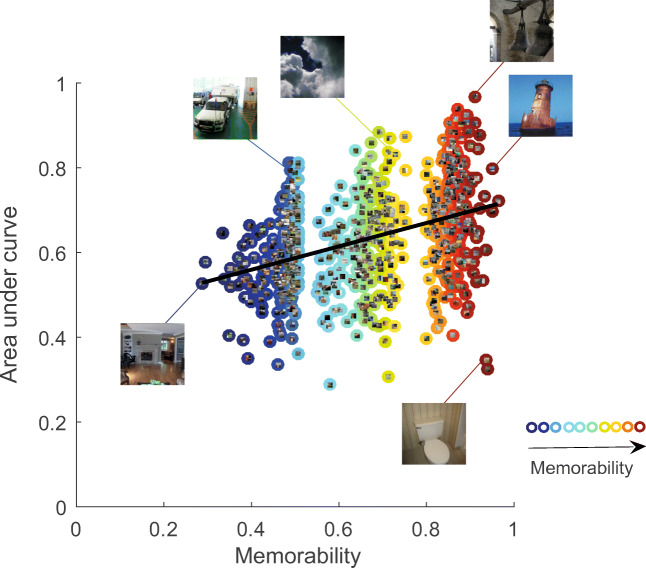
Area under the curve per scene. Area under the curve was strongly positively associated with increasing memorability score. Each *circle* in the figure represents an image

Across subjects, recognition performance was better for images in the high-mem category than for the mid-mem category, as indicated by higher hit rates (*t*(44.00) = 7.61, *p* < 0.001, *d* = 1.13), lower false-alarm rates (*t*(44.00) = − 4.81, *p* < 0.001, *d* = − 0.72) (see Table 1), and higher *d’* (*t*(44.00) = 10.28, *p* < 0.001, *d* = 1.53). Likewise, hit rates (*t*(44.00) = 4.34, *p* < 0.001, *d* = 0.65) and *d’* (*t*(44.00) = 3.07, *p* = 0.004, *d* = 0.46) were higher for images in the mid-mem category than for the low-mem category, but false-alarm rates did not differ between these categories (*t*(44.00) = 0.35, *p* = 0.725, *d* = 0.05). Moreover, area under the ROC curves (AUC) was strongly positively associated with memorability (Spearman’s *ρ* = 0.41, *p* < 0.001) across images (see Fig. 4). Across subjects, AUC was larger for the high-mem category than for the mid-mem category (*t*(44.00) = 10.50, *p* < 0.001, *d* = 1.57). Likewise, AUC was larger for images in the mid-mem category than for the low-mem category (*t*(44.00) = 3.44, *p* = 0.001, *d* = 0.51).

#### ROC and model results

**Fig. 5:**
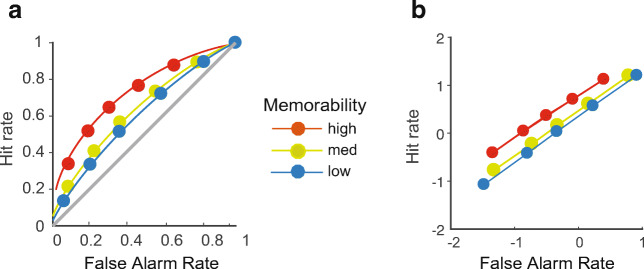
Results Experiment 1. **a** ROC-curves are largely curvilinear, a prediction made by the UVSD model. They are bent upwards for more confident decisions and with larger area under the curve for highly memorable images, indicating more memory strength. **b** z-ROCs are linear with a larger intercept for highly memorable images

ROCs had a curvilinear shape whereas zROCs were linear, which are shapes better predicted by the UVSD model (Fig. 5). Accordingly, the *G* statistic confirmed that single subject data were successfully fitted by the UVSD model for 85% of participants, while the DPSD successfully fitted the data of only 70% of participants. The aggregate and individual participant data were better fitted by the UVSD model than by the DPSD model, indicated by lower BICs for the UVSD model across all participants. The sensitivity parameter *d’* of the UVSD model was significantly larger for the high-mem compared to the low-mem category (*t*(44.00) = 10.50, *p* < 0.001, *d* = 1.57). In contrast, the parameter modeling the variance of the old item distribution *Vo* was not significantly different between the two categories (*t*(44.00) = 1.76, *p* = 0.089, *d* = 0.26). Both the recollection (*t*(44.00) = 4.94, *p* < 0.001, *d* = 0.74) and the familiarity parameter (*t*(44.00) = 7.61, *p* < 0.001, *d* = 1.13) of the DPSD model were larger for high-mem compared to low-mem images.

### Discussion experiment 1

Overall, the results replicate previous studies showing that intrinsic image memorability is a robust feature of an image, which affects people’s memory performance independently of personal factors. The ROC analysis confirmed and extended previous studies of memorability, which had focused on hit rates, by showing that memorable images also yield larger AUC.

The ROC curves were better fitted by the UVSD model, which assumes that recognition is based on a single, continuous memory strength dimension. The superiority of the UVSD appears plausible given the symmetrical, curvilinear shapes of the ROCs. Greater memorability was associated with larger sensitivity (*d’*), but not with greater variability of the old item distribution (*Vo*). While this model does not deny that some conditions, e.g., recognition of highly memorable images, tend to coincide with recollection of specific details associated with the studied item, it treats recollection simply as reflecting higher memory strength. Hence, in this experiment recognition was not based on a specific recollection process independent of memory strength, as predicted by the DPSD model. This finding could imply that recognition of scene images is generally based only on memory strength and that the superior recognition performance for highly memorable images is not associated with a separate recollection process.

However, the specific shape of the ROC curves in Experiment 1 might also be due to the overall low performance in the recognition task. Indeed, hit rates were consistently lower in our study than hit rates obtained for the same images in previous studies. Performance in our study might have been affected by the specific memory task: most previous memorability studies (Isola et al., 2014; Bylinskii et al., 2015) used continuous recognition tasks where the delay between encoding and test is shorter and the number of intervening items is significantly smaller compared to a design with separate encoding and testing blocks. In addition to this inevitable difference, other more amendable factors might have been responsible for the poor performance as well. First, presentation durations (500 ms) were shorter than in previous studies (1000 ms and 2000 ms in Isola et al., 2011 and Bylinskii et al., 2015). Second, participants had to perform an additional indoor/outdoor discrimination task. Together, these factors may have contributed to shallow rather than deep encoding of image aspects, thus obstructing the potential for recollection. Furthermore, the recognition task in the test phase, which required only a simple old/new decision instead of a report of the recollective experience, may have encouraged participants to base their recognition decisions and confidence judgments more on memory strength than on recollection.

In order to substantiate the association of recollection and intrinsic image memorability (or the lack thereof) we conducted a second experiment, in which encoding was facilitated and recognition required an additional judgment of recollective experience.

## Experiment 2

Experiment 2 was similar to Experiment 1 with a few modifications. Most importantly, participants were to report their recollective experience with R/K judgments (Tulving, 1985). The R/K judgments were introduced to acquire an additional index of recollection independent of model parameters derived from ROC curves.

A hallmark finding regarding R/K judgments has been obtained in a study, in which words were learned under deep versus shallow encoding or full versus diverted attention conditions (Yonelinas, 2001). Results showed a perfect crossover: the proportion of deeply encoded and fully attended words was greater among remember statements whereas words presented in the shallow and diverted attention condition were more associated with know statements. Moreover, Tsivilis et al., (2001) studied R/K statements with picture stimuli and found that the proportion of R statements increased if to-be-remembered objects are presented in their original scene contexts, whereas the proportion of K statements was unaffected by object context.

However, using R/K judgments as an accurate index of recollection or familiarity is anything but trivial due to procedural (Migo, Mayes, & Montaldi, 2012) and statistical (Yonelinas, 2001; Haaf et al., 2020) challenges. First, if not instructed carefully, participants might confuse the “remember” category simply with high confidence, neglecting that a feeling of familiarity can occasionally go along with high confidence, too. Therefore, we followed recommendations for R/K procedures put forward by Migo et al., (2012, see Methods/Procedure). Second, the statistical analyses must account for the fact that the proportions of R and K statements are interdependent. Specifically, the probability of a know response is mathematically constrained by the proportion of remember responses and vice versa, making inferences assuming their independence (as in Gardiner & Java, 1990) statistically inappropriate (see Yonelinas, 2001 ). Therefore, we applied an analysis framework proposed by Haaf et al., (2020) (see Methods/Analysis).

Moreover, we extended the conventional remember/know framework by additionally asking for analogous judgments for new items, thereby exploring the mnemonic experience associated with the rejection of new information. Thus, whenever participants decided that an item was new, we asked whether they considered specific image details (D judgment) to be relevant for their decision or whether the item simply felt unfamiliar (U judgment). The D/U judgments for new items are thus equivalent to R/K judgments for old items and were thus analyzed with the same analysis framework.

### Methods

Unless otherwise specified, the procedures used in Experiment 2 were identical to Experiment 1. All procedures and analyses were conducted as preregistered unless stated otherwise (see Open Practices Statement).

### Participants

Fifty participants (46 female, mean age = 21.24), none of whom had participated in the first study, were recruited from the University of Muenster community. Participation was compensated with course credit. To determine our sample size, we followed the same reasoning as (Haaf et al., 2020) in their effort to replicate (Gardiner and Java, 1990). Specifically, Haaf et al could not reproduce the original findings with twice the statistical power, implying the possibility that the original R/K finding is a false-positive. To have the same statistical power in our data, we more than doubled the sample size and amount of trials compared to the original R/K experiment by Gardiner and Java (1990). In keeping with the criteria described in the preregistration, eight participants whose performance was no better than chance were excluded from analysis.

### Apparatus and materials

Stimuli were shown on a 19-inch CRT monitor with 1280x768 resolution and a 60-Hz refresh rate, using a PC with a 2.53 GHz Intel Core processor and 8 GB RAM, running a Windows 10 64-bit operating system. We selected 360 pictures from the memorability image database *FIGRIM* (Bylinskii et al., 2015), shrunk to a resolution of 500x500 *px* (120 images per memorability category). For Experiment 2, we only selected images from the FIGRIM database because it contains more exemplars per semantic category, allowing for a more balanced stimulus set. Specifically, we selected images from only 14 semantic categories (as compared to 241 semantic categories in Experiment 1), each comprising 4 to 16% of the total stimulus set (see Table 6 and Fig. 9 in the Appendix for the distribution of semantic categories across the stimulus-set and across memorability scores, respectively). Again, we counterbalanced indoor/outdoor scene gist across memorability categories. Some of the selected semantic scene categories had only very few high-mem exemplars (e.g., highway) or low-mem exemplars (e.g., playground). In order to counterbalance indoor/outdoor scene gist and to maximize the number of trials for each memorability bin and semantic category, we had to make minor adjustments for some categories to the boundary between the low-mem and mid-mem, and between the mid-mem and high-mem bin, respectively. For example, while for most scene categories the boundary between mid-mem and high-mem was a memorability score of 0.75, one of the most memorable highway images had a memorability score of only 0.74, making us lower the boundary to 0.74 for the highway category. Although such an adjustment was necessary for only few categories and few images, it slightly blurred the distinction between low-mem and mid-mem, and between mid-mem and high-mem bins. Therefore, we chose to conduct statistical comparisons only between the hi-mem and lo-mem bin, which were clearly non-overlapping for all scene categories.

Half of all images were presented at the encoding phase, while the other half served as foils for the test phase. Thus, Experiment 2 comprised fewer items than Experiment 1, which was necessitated by the increased presentation durations. However, we followed recommendations by Yonelinas and Parks (2007) who argued that 120 trials (60 old, 60 foil pictures) are necessary for reliable ROC-curves.

### Procedure

**Fig. 6:**
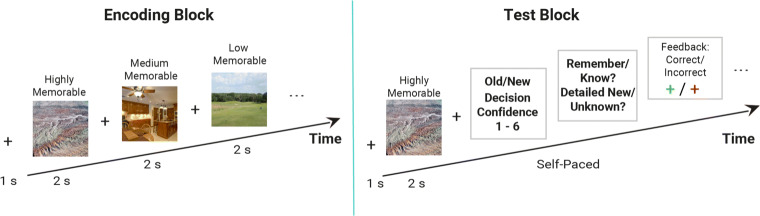
Illustration of a trial sequence. Encoding Block: Each trial starts with a fixation cross for 1 s, followed by a picture presented for 2 s and a blank screen for 200–500 ms, repeated 180 times. Test block: Each trial starts with a fixation cross presented for 1 s, followed by a picture presented for 2 s and participants had to decide with a button press whether the picture was old or new and how confident they were about their decision on a scale from 1 (sure new) to 6 (sure old). If they pressed 1–3 (“old”), they had to indicate afterwards whether they remembered or just knew the scene. If they pressed 4–6 (“new”), they had to indicate whether the scene was simply unfamiliar to them (“it feels new, but I do not know why”) or whether particular details in the scene were particularly new to them

The procedure was identical to that of Experiment 1 except for the following notable changes (see Fig. 6). First, the presentation duration was increased to 2000 ms in both the encoding and test phase. Second, the indoor/outdoor discrimination task was removed from the encoding phase. Most importantly, additional R/K and D/U judgments were required on each trial of the test phase.

In the encoding block, trials started with a fixation cross (1000-ms duration), followed by a scene image (2000-ms duration), followed by a blank screen for a random interval between 200 and 500 ms. In the test block, trials started with a fixation cross (1000-ms duration), followed by a scene image (2000 ms), followed by a blank screen with a random interval between 200 and 500 ms, followed by response prompts. Participants made an old/new judgment accompanied by a confidence judgment (from 1/sure new to 6 /sure old), and finally made either a R/K statement (for items judged as old) or D/U (for items judged as new) statement. Note that the confidence range did not change with respect to Experiment 1 but that old/new and confidence judgments were collapsed on the same scale to make the procedure as efficient as possible 2.

Instructions for R/K statements emphasized recommendations made by Migo, Mayes, and Montaldi (2012) to accentuate the distinction between recollection and familiarity. Specifically, know statements should be based on a feeling of familiarity for the scene, without any contextual knowledge about the encoding period. Remember statements on the other hand should be based on recollection of specific image aspects and the original encoding context. To this end, we carefully explained the concept definitions of R/K statements. We also emphasized that R statements do not need to refer to one particular object or feature, but could also refer to multiple objects/features/image parts. We emphasized that K statements can equally be based on high or low confidence in order to avoid a bias towards remember statements in states of high confidence.

Finally, instructions for D/U statements were explained to be a conceptual counterpart of R/K statements. Specifically, detailed-new statements were supposed to be based on any image aspects that participants particularly considered for their new-decision. Unfamiliar statements were simply based on the feeling that a particular image is new, no matter how certain participants were in their judgment.

After practice trials, participants had to explain the instructions back to the experimenter to make sure that they really understood the procedure. Lastly, in 2 to 4 (depending on the frequency of remember statements) out of 360 trials, participants were asked via a prompt to motivate their remember statement in a short sentence.

### Analysis

Given that a “know” statement implies a “not remember” statement, and vice versa, the proportions of R/K and D/U statements are interdependent. (Haaf et al., 2020) put forward a scaled difference metric, which integrates the proportions of R and K statements into a single outcome measure in a way that accommodates their dependency. Thereby, the scaled difference avoids a flaw in the analysis by Gardiner and Java (1990) who treated R and K statements as independent of each other, and analyzed statement-type as an ANOVA factor, i.e., as independent (manipulated) variables instead of a dependent (outcome) variable. For items judged as old, the scaled difference *Yold* for the *i* th participant and *j* th memorability condition is defined as:

$$ Yold_{ij} = \frac{r_{ij} - k_{ij}}{r_{ij} + k_{ij}} $$

where r and k indicate the proportions of remember and know statements, respectively. Likewise, for items judged as new, the scaled difference *Ynew* is defined as:

$$ Ynew_{ij} = \frac{d_{ij} - u_{ij}}{d_{ij} + u_{ij}} $$

where d and u indicate the proportions of detailed and unfamiliar statements, respectively. The scaled difference score is positive when the proportion of old-remember or new-detailed statements is larger than the proportion of old-know or new-unfamiliar statements, respectively. A scaled difference score of zero indicates no propensity for either remember/new-detailed or know/unfamiliar.

We hypothesized that highly compared to low memorable images are more associated with remember/detailed-new rather than know/unfamiliar statements whereas the null hypothesis predicts no difference in scaled differences between the two memorability conditions.

Given the relatively poor performance of the DPSD model and the curvilinear shapes of the ROCs in Experiment 1, we adopted a Bayesian analysis framework to test the evidence for these hypotheses (Morey & Rouder, 2011). Specifically, we analyzed Bayes factors, which are likelihood-ratio tests comparing the likelihood of the data under the null hypothesis with the likelihood of data under the alternative hypothesis:

$$ BF_{01} = \frac{likelihood of data given H_{0}}{likelihood of data given H_{1}} $$

Importantly, unlike conventional frequentist inferential tests such as the *t* test, a Bayes factor analysis allows quantifying the evidence in favor of the null hypothesis, relative to evidence for the alternative hypothesis.

To test the evidence for our hypotheses and for the null hypotheses, respectively, we used one-sided Bayesian *t* tests, adopting the terminology proposed by Jeffreys (1961), with a Bayes factor larger than 10 suggesting strong evidence for the alternative hypothesis and a Bayes factor equal to or smaller than 1/10 meaning strong evidence for the null hypothesis. As exploratory analyses, we also tested whether scaled differences for the high memorability condition differ from zero (meaning no propensity for either recollection/detailed-new or know/unfamiliar statements). Furthermore we quantified *Ynew* and *Yold* for each image and correlated these scores with the images’ memorability. 3 Behavioral analyses and Bayes factor analysis were conducted with the R programming language in the RStudio environment (R Core Team, 2014; Team & et al. 2015) using the BayesFactor package developed by Morey, Rouder, Jamil, and Morey (2015). Effect sizes of these analyses are reported as Cohen’s *d* (Cohen, 1988), computed according to (Lakens, 2013).

ROC curves were fitted with a DPSD model (Yonelinas, 1994) and a UVSD model (Mickes et al., 2007) using ROC Toolbox for MATLAB by Koen et al., (2017). We first considered whether the models generally provide a statistically acceptable account of the data based on the *G* statistic (McDonald, 2009; Koen et al., 2013). We then compared model performance on the basis of the Bayesian Information Criterion (BIC; Schwarz et al. 1978; Lewandowsky & Farrell, 2010). The model with lower BICs in 80% of participants was declared the winning model under the condition that the model has a statistically acceptable account of the data in at least 80% of participants. Given the model results of Experiment 1, we predicted better performance by the UVSD model compared to the DPSD model.

However, given that the UVSD model is more flexible in fitting a wider range of ROC curves (Klauer & Kellen, 2011), we complemented our preregistered model comparison process by investigating how well each set of model parameters predicts item memorability ranks in a separate regression model.^4^ More specifically, we rank ordered images from lowest to highest memorability and built 30 quantiles of equal trial numbers, yielding sufficient power to fit ROC curves per memorability quantile. We then fitted both the UVSD and the DPSD model for each quantile and used the recovered parameters to predict memorability quantile ranks using an ordinal regression model (Harrell Jr, 2015). Finally, we considered for each model how much variance in memorability ranks was explained by its set of parameters (*R*^*2*^ *Adjusted*). Additionally, we tested how much each model parameter contributed to the explained variance by considering their standardized coefficients.

## Results experiment 2

### Recollective reports

We categorized a total of 162 verbal reports following a remember statement. In 36% of all reports, participants exclusively reported specific objects and/or scene details as part of their recollective experiences (e.g., “I remember this orange coffee mug”). Another 47% of all reports included additional associations between image aspects and personal thoughts or experiences (e.g., “The stop lights are on and I was wondering whether he caused a traffic jam or an accident”), autobiographical memories (e.g., “The picture reminded me of a photograph that I took during vacation”), or evaluative judgments (“Looks very bleak, like an insufficiently furnished student apartment”). Only 9% of all reports explicitly refer to distinctive or unusual image details. Two participants reported that they knew a depicted building because it was a famous site (London Tower Bridge). We removed this picture from further analysis. Three participants reported that they knew a scene because they had been there before on vacation (badlands scene from Alberta, Canada and Petronas Twin Towers in Kuala Lumpur, Malaysia). We removed these trials from further analysis.

### R/K scaled differences for hits and false alarms

As can be seen in Table 2, performance was substantially better in Experiment 2, with larger hit rates and lower false-alarm rates compared to performance in Experiment 1, see Table 1. Independent two-sample *t* tests confirmed that participants had significantly larger hit rates (*t*(84.47) = 5.65, *p* < .001) and significantly smaller false-alarm rates (*t*(81.81) = − 4.35, *p* < .001) in Experiment 2 compared to Experiment 1.

**Table 2:** Mean memorability scores as well as mean hit rates and false-alarm rates from Experiment 2 per memorability category and indoor/outdoor scene gist

Memorability	Scene gist	Memorability score	Hit-rate Exp 2	False-alarm rate Exp 2
High	Indoor	0.83	0.76	0.22
High	Outdoor	0.82	0.76	0.24
Medium	Indoor	0.62	0.66	0.24
Medium	Outdoor	0.64	0.67	0.25
Low	Indoor	0.45	0.60	0.19
Low	Outdoor	0.44	0.54	0.18

**Fig. 7:**
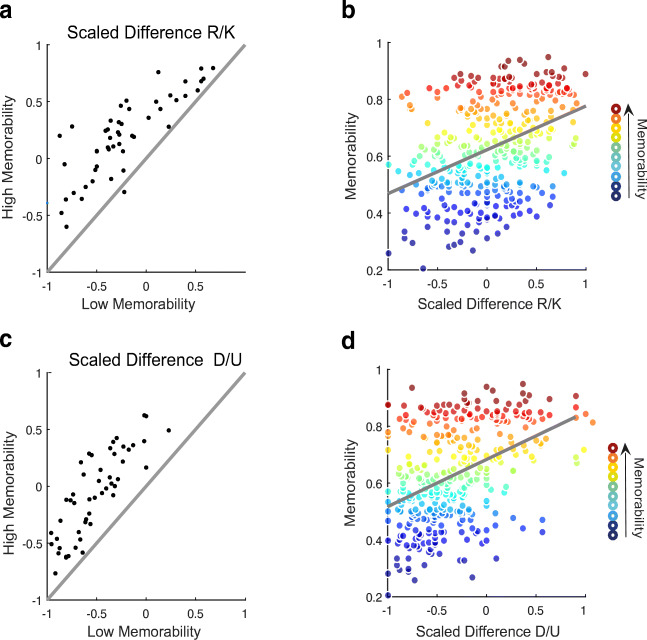
Results experiment 2. **a** R/K scaled differences for correctly recognized old items for high memorability mapped against low memorability, each *dot* is the score of one subject. If dots scattered around the line, no difference in conditions would be assumed. The great majority of dots lies above the line and above 0, meaning that subjects had a greater propensity for remember responses in highly vs. low memorable images. **b** Correlation between memorability scores per picture and scaled difference for correctly recognized old items, each dot represents an image. **c** D/U scaled differences for correctly rejected new items for high memorability mapped against low memorability, each *dot* is the score of one subject. The great majority of dots falls above the line but the average is slightly below zero. Participants had thus a more positive propensity towards detailed new judgements for highly memorable images but the majority scored below zero. **d** Correlation between memorability scores per picture and scaled differences for correctly rejected new items, each *dot* represents an image

Participants made a greater proportion of R statements for high-mem images and a greater proportion of K statements for low-mem images (see Table 3). A one-sided Bayesian *t* test yielded extreme evidence for the alternative hypothesis that participants were more likely to recollect high-mem compared to low-mem images (*BF* = 3477.914, *d*= 1.03; see Fig. 7a). Overall, *Yold* scores of high-mem images were positive, providing extreme evidence that highly memorable images are associated with remember rather than know statements (*BF* = 206.03, *d* = .87). As an exploratory analysis, we correlated the *Yold* score per picture with its memorability score and we found a moderately strong relationship between the two variables (*r*(357) = .38, *p* < .001). Nonetheless, as can be seen in Fig. 7B, there is considerable variability in scaled differences for highly memorable images.

**Table 3:** Response proportions and scaled differences per memorability level

Mem	R	K	*Yold*	D	U	*Ynew*	R_*f*_	K_*f*_	*Yold*_*f*_	D_*f*_	U_*f*_	*Ynew*_*f*_
H	0.61	0.39	0.23	0.47	0.53	-0.04	0.14	0.86	− 0.72	0.33	0.67	− 0.35
M	0.52	0.48	0.04	0.35	0.65	-0.30	0.10	0.90	− 0.80	0.24	0.76	− 0.51
L	0.43	0.57	− 0.13	0.75	0.25	-0.72	0.08	0.92	− 0.85	0.14	0.86	− 0.72

Abbreviations: R = Remember, K = Know, *Yold* = R/K Scaled Difference, D = Detailed-New, U = Unfamiliar, *Ynew* = D/U Scaled Difference, R_*f*_ = Remember statements based on false alarms, K_*f*_ = Know statements based on false alarms, *Yold*_*f*_ = R/K Scaled Difference based on false alarms, D_*f*_ = Detailed-New statements based on false rejections, U_*f*_ = Unfamiliar statements based on false rejections *Ynew*_*f*_ = D/U Scaled Difference based on false rejections

For false alarms, *Yold* scores were negative for both memorability categories, reflecting a greater proportion of “know” judgments compared to “remember” judgments.*Yold* scores were slightly less negative for high-mem (mean *Yold*: -.77) than for low-mem (mean *Yold*: -.90) images, indicating a slightly stronger bias for false “remember” statements for hi-mem images. However, a Bayesian *t* test indicated only weak to moderate evidence of a real difference in *Yold* scores between memorability categories compared to the null hypothesis of no difference (*BF* = 3.828, *d*=.49).^5^

### D/U scaled differences for correct and incorrect rejections

Participants made more D statements for high-mem images than for low-mem images, but they did not on average prefer D over U statements (Table 3). A one-sided Bayesian *t* test contrasting the high-mem with the low-mem condition yielded extreme evidence for the hypothesis that an increase in memorability is associated with an increase in D statements (*BF* = 1032293, *d*= 1.36)(see Fig. 7b). A one-sided Bayesian *t* test testing scaled differences for highly memorable images against zero revealed strong evidence for the null hypothesis that participants had no overall propensity for either D or U statements (BF = .085, *d*= 0.24). As an exploratory analysis, we correlated the *Ynew* score per picture with its memorability score and we found a moderately strong relationship between the two variables (*r*(357) = .40, *p* < .001)(see Fig. 7d). Correlating *Yold* and *Ynew* scores revealed a strong relationship between the two measures (*r*(357) = .67,(*p*) < .001).

For new images incorrectly reported as old, a Bayesian *t* test yielded extreme evidence that scaled differences were less negative for high-mem than for low-mem images (*BF* = 12089.17, *d*=.1.10). This means that even when participants falsely judged a high-mem image to be new, they were more likely to identify image details to be relevant for their decision.

### ROC and model results

**Fig. 8:**
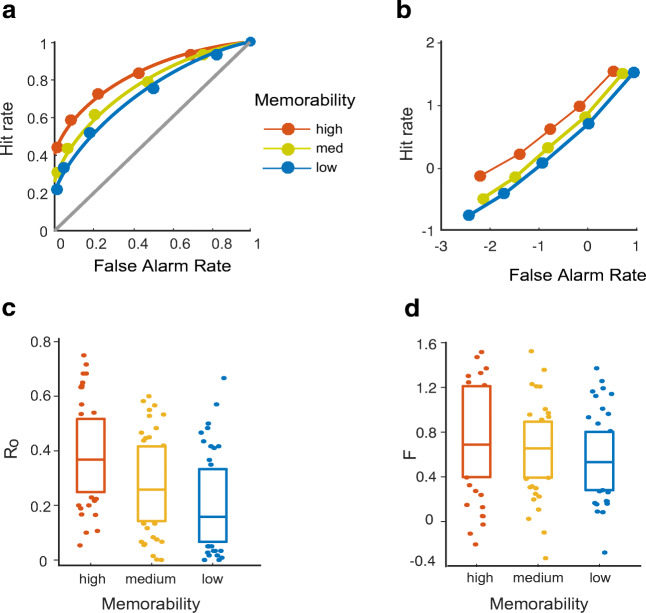
ROC and model results experiment 2. **a** ROC-curves are more hockey-stick-shaped for highly memorable images, bent upwards for more confident decisions and with larger area under the curve, indicating more recollection and familiarity for highly memorable images. **b** z-ROCs are bent upwards for most conservative responses, suggesting increased recollection. **c** Recollection parameter in the DPSD model is larger for highly compared to low memorable images. **d** Familiarity parameter in the DPSD model is larger for highly compared to low memorable images

**Fig. 9:**
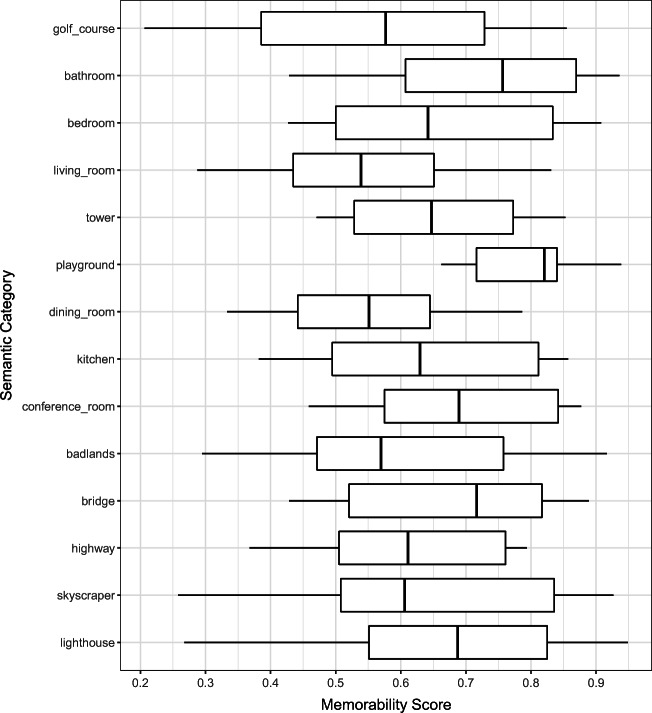
Distribution of memorability scores across semantic categories, Experiment 2

ROC curves of single subjects and of the aggregate data were fitted with a DPSD model and the UVSD model. Visual inspection of ROC curves supports the results of our Remember/Know procedure (Fig. 8a and b): for highly memorable images, z-ROCs are bent upwards for more conservative responses and ROC curves are asymmetric, visually indicating increased recollection for memorable pictures. Model results reveal that both models successfully fitted the data in more than 80% of individual participant data, based on the *G*-statistic. The data were better fitted by the DPSD model than by the UVSD model in the aggregate data and in 60% of single participant data, indicated by lower BICs for the DPSD model. However, we could not determine a winning model according to our preregistered criterion of a best fit in 80% of participants.

Nonetheless, memorability quantile ranks were predicted better by the ordinal regression model that included DPSD parameters (*χ*^2^ = 47.67, *p* =.001, *R*^*2*^ *Adjusted* = 78%) than the regression model with UVSD parameters (*χ*^2^ = 42.78, *p*< .001, *R*^*2*^ *Adjusted* = 76%). For the DPSD model, the recollection parameter contributed more to the regression’s explained variance (*β* = 3.31, *SE* = 0.687, *Z* = 4.82, *p* < .001) than the familiarity parameter (*β* = 0.904, *SE* = 0.436, *Z* = 2.07, *p* = .038). For the UVSD model, only *d’* contributed significantly to the regression’s explained variance (*β* = 3.9904, *SE* = 0.802, *Z* = 4.97, *p* < .001), whereas the *Vo* parameter indicating old item variance did not significantly contribute to the model (*β* = − 0.695, *SE* = 0.493, *Z* = − 1.41, *p* = 0.158). This was confirmed by simple t-tests comparing each model parameter between high and low memorable images. While both parameters showed an effect of memorability, the effect was much stronger on the recollection parameter Ro (*t*(41) = 9.44, *p* < .001; see Fig. 8c) than on the familiarity parameter F (*t*(41) = 2.63, *p* = .01; see Fig. 8d).

### Discussion experiment 2

The results of Experiment 2 showed that intrinsic image memorability boosts recollection. Specifically, hi-mem images were more often recognized based on recollection than on familiarity, while low-mem images were more often recognized based on familiarity than on recollection. There was no corresponding effect of memorability on false alarms: false recollection of new images was only slightly more prevalent for hi-mem than for low-mem images. Thus, the memorability-related boost in recollection for old images cannot be explained by an effect of memorability on response criterion (Haaf et al., 2020). In sum, image memorability is not purely a measure of increased familiarity (i.e., “I think I have seen this amusement park scene before”) but is also more likely to invoke episodic associations regarding specific image aspects (e.g., “I recognize this amusement-park scene because I thought this roller-coaster is so steep”). This interpretation is supported by the recollection reports, which included a large number of references to specific image content.

Our results resemble the “crossover effect” typically observed in R/K studies. For instance, Yonelinas (2001) showed a greater proportion of remember responses for deeply encoded and fully attended words and a greater proportion of know responses in shallow encoding as well as diverted attention conditions. This pattern of results has been interpreted as evidence in favor of dual-process models of recognition memory (Yonelinas, 2002; Haaf et al., 2020). While similar in spirit to the studies by Yonelinas (2001) and Haaf et al., (2020), the present study introduced two notable modifications that may be responsible for finding a crossover effect. Firstly, participants in our study expected to justify their remember statements at least in a subset of trials. This procedural difference corroborates recent findings emphasizing the importance of motivating remember statements to avoid a potential confound with mere memory strength (Migo et al., 2012). This line of reasoning supports the conclusions made by Haaf and colleagues: the original R/K findings by Gardiner and Java may not be reproducible, given that participants did not need to justify their remember statements. Secondly, while certain experimental manipulations (e.g., the lexical status of a word) might not have a robust differential effect on recollection and familiarity, intrinsic image memorability might be a more promising candidate.

We complemented the conventional remember/know procedure, in which remember/know judgments are only made for items recognized as old, with equivalent detailed/unfamiliar judgments for items rejected as new. We found that image memorability not only boosted the proportion of remember judgments for old items but also the proportion of detailed-new judgments (i.e., “I would have remembered this object if I had seen this image before”) for new stimuli. Moreover, both judgments were correlated across images, meaning that images that are recollected when old also tend to be rejected based on image details when new. The description of our detailed-new category resembles the description of a recollect-to-reject process. For instance, Yonelinas (1997) reasoned that “after studying a short list of words, one would likely not false alarm to one’s own name if it appeared in the test list” (p. 752). Likewise, it stands to reason that in a visual memory experiment subjects would also not false alarm to an image of their own house (“I would remember if I had seen my own house”). While the present study was not designed specifically to test recollection to reject new items, it is interesting to compare these lines of research. A typical procedure for studying recollect-to-reject processes is associative recognition, where subjects learn lists of word-pairs (tree-shoe) and are tested with the original word pairs and rearranged foils (e.g., tree-dog). A related procedure is the plurality recognition task, where subjects learn lists of singular and plural words and are tested with the original plurality (e.g.,“frog”) or the reversed plurality (“frogs”). Studies analyzing the shapes of ROC curves (Yonelinas, 1997) and response time distributions (Rotello and Heit, 1999; 2000) found that subjects not only could recollect that original items had been on the study list, but occasionally recollected that new items had not been studied. An intuitive explanation for this finding is that on some trials, a foil item like “tree-dog” (or “frogs”) can evoke the recollection of having learned “tree-shoe” (“frog”). Interestingly, and in contrast to Yonelinas’ illustrative example, these studies have found no evidence for a recollect-to-reject process in simple item recognition tasks that are equivalent to our image recognition task.

However, in spite of the apparent similarity between the detailed-new response category and recollection to reject, there is reason to doubt that they correspond to the exact same mnemonic process. First, previous studies have inferred recollect-to-reject processes from ROC curves and response time distributions, but have not required subjects to make detailed-new vs. unfamiliar statements (i.e., the equivalent of remember/know statements), making our paradigm difficult to compare. Second, as just described, previous studies introduced for each new foil item a corresponding original old item, for which the foil could trigger a recollection. Our study did not have similar corresponding original and foil items; it is unlikely that judging that “I would remember if I had seen this playground before” is actually based on recollecting all the other playground images from the study phase. Even if that were the case, then the probability of a detailed-new response to a new image should have been determined by the memorability of these other studied images. By contrast, we found that the proportion of detailed-new responses was strongly determined by the new image’s own memorability. Finally, recollection of new items in associative recognition or plurality tasks requires that the foils be similar to their associated original item to trigger the old item’s recollection. By contrast, highly memorable images, for which we found the highest proportion of detailed-new responses, are particularly dissimilar from other images (Bylinskii et al., 2015; Lukavskẏ & Děchtěrenko, 2017). In sum, it is not clear if detailed-new reports in our study are equivalent to recollect-to-reject processes found in previous studies, or if findings from experiments using word stimuli are generalizable to experiments using scene images.

Interestingly, the analysis of remember/know and detailed-new/unfamiliar judgments associated with individual images also revealed a great deal of variability even at the same level of memorability. Especially at high memorability, some images were almost exclusively judged as remembered when recognized and as new-detailed when rejected, while almost as many others were predominantly judged as known and unfamiliar, respectively. Only the least memorable images were predominantly judged as familiar and unfamiliar, respectively (see Fig. 7b and d). This finding shows that the strong relationship between memorability and recollection is not unitary across images: some images actually achieve high recognition rates by selectively boosting familiarity.

Performance in Experiment 2 was substantially better than in Experiment 1, with both higher hit-rates and lower false-alarm rates. This suggests that the modified task and procedures allowed for better encoding and more recollection of specific image aspects. First, we almost halved the number of images participants had to memorize, decreasing the overall memory load. Previous memorability work largely utilized continuous recognition tasks where the number of items between encoding and test is significantly smaller compared to a design with separate encoding and testing blocks. Secondly, in Experiment 2 participants were allowed to simply view and encode the images, whereas in Experiment 1 participants performed an indoor/outdoor gist discrimination task while encoding the images. Previous research has shown that memory performance is strongly impaired when attention is divided between encoding and a concurrent task (Naveh-Benjamin, Guez, & Sorek, 2007). Moreover, the frequency of recollective experiences is reduced under divided-attention conditions at test (Jacoby, 1991). Lastly, picture presentation times were quadrupled from 0.5 to 2 s in the encoding block and doubled from 1 to 2 s in the testing block. Ahmad, Moscovitch, and Hockley (2017) showed that an increase in presentation time increased memory performance based on perceptual details of an image. Hence, by reducing the number of to-be-remembered stimuli, dropping the dual-task requirement during encoding and increasing the presentation time at both encoding and testing in Experiment 2, more memory and attentional resources could be allocated to encoding of specific image details for subsequent recollection.

It is important to mention that not only recollection but memory performance overall improved substantially from Experiment 1 to Experiment 2, which has direct consequences for our model selection process, given that the two experiments favor different models. In contrast to Experiment 1, ROC curves were not well fitted with the single-process UVSD model; the overall shapes of ROC curves were visually and quantitatively more consistent with the DPSD model. As a case in point, ROCs in z-space were curved (see Fig. 8b), which is a finding typically observed in relational recognition tasks where participants have to judge not only whether an item is old but also whether it occurred in a specific encoding context (e.g., as member of a list or a word pair)(Yonelinas, 1997). Importantly, curved ROCs in z-space are a prediction specifically made by dual-process and not single-process accounts (Yonelinas & Parks, 2007). In addition, the DPSD model outperformed the UVSD model in the aggregate data, but only in 60% of individual participants. However, the superiority of the DPSD model was also supported by an exploratory (i.e., not preregistered) analysis in which we used ordinal regression to predict the images’ memorability based on the DPSD and UVSD model parameters, respectively. Although the difference in explained variance (e.g., 2%) was not large, memorability was best predicted by DPSD model parameters, where the recollection parameter made larger contributions to the regression model compared to the familiarity parameter. In the regression based on UVSD-parameter, the parameter indicating old item variance did not contribute to the regression model, corroborating the findings from Experiment 1.

Even though the model comparison was not as unequivocal as anticipated in the preregistration and unlike the results of Experiment 1, the results of Experiment 2 clearly indicate that memorability boosts recollection, arguable due to the overall better overall performance in Experiment 2. This conclusion is supported by the R/K judgments, the ROC model comparison, and our exploratory regression analysis. In sum, the results strongly support the notion that higher memorability is specifically associated with a higher incidence of recollection.

## General discussion

Previous research has defined memorability exclusively in terms of objective recognition performance (i.e., hit rates), which can be predicted with machine vision algorithms (Isola et al., 2014; Khosla et al., 2015; Peng et al., 2015). However, the features making an image memorable and the cognitive mechanisms affected by these features are still elusive. Although it is reasonable to assume that there is something special and subjectively remarkable about memorable images, subjective ratings of interestingness are only poorly correlated with memorability and observers actually cannot accurately judge which images are memorable (Isola et al., 2014). Memorable images do not differ from non-memorable images in terms of low-level image statistics (Isola et al., 2014) and they do not differentially activate early visual cortex (Bainbridge, Dilks, & Oliva, 2017; Bainbridge & Rissman, 2018) but they are more easily perceived at ultra-fast presentation times (Broers et al., 2018). Furthermore, Bainbridge (2020) found that the difference between memorable and non-memorable images is not due to more elaborate encoding, stronger attentional capture, or stronger motivation to remember an image. Lastly, an image’s memorability is only moderately determined by its distinctiveness relative to other images shown in the same experiment: an image’s memorability is largely preserved whether it is one of few exemplars of its semantic category, or one of many (Bylinskii et al., 2015). Instead, memorability is correlated with specific semantic image content: images of social activities, faces, human-made objects, animals, and interiors are on average recognized better than panoramic views of nature. However, each of these scene categories comprises a full spectrum ranging from highly memorable to forgettable, indicating that memorability cannot be exclusively explained by semantic image category (Bylinskii et al., 2015).

Which psychological mechanisms are affected by memorable image features; which mechanisms are responsible for their improved recognition? A common thread in the previous literature (with the exception of Bainbridge, 2020) has been a focus on image features correlated with memorability, but not on the cognitive processes involved in remembering such images. The present study aimed to contribute to the latter question by investigating how intrinsic image memorability affects recollection and familiarity. The results of our remember/know (R/K) procedure in Experiment 2 revealed that, on average, memorability specifically boosts recollection, indicating that intrinsic image memorability affects both objective (i.e., hit rates) and subjective (i.e., R/K judgments) indices of recognition memory consistently across people. Interestingly, a more fine-grained analysis revealed that, even at a given level of memorability, there is considerable variability across images in how they are remembered, especially for highly memorable images: while some images were recognized almost exclusively based on recollection, others were mostly recognized based on familiarity. It would be interesting for future work to investigate which image content determines an image’s potential for recollection or familiarity.

While the R/K judgments revealed a clear effect of memorability on recollective experience, the results of the ROC analysis were more ambiguous regarding the nature of the underlying cognitive mechanisms. ROC curves in Experiment 1 were largely curvilinear and were better fitted, in all participants, by a single-process model. By contrast, In Experiment 2, ROCs and z-ROCs visually indicated recollection and were better fitted by a DPSD model, albeit in only 60% of participants. However, DPSD model parameters performed better in an additional regression model. Here, memorability was best predicted by DPSD model parameters, and the recollection parameter made larger contributions to the regression model compared to the familiarity parameter. Given that the additional regression analysis was not included in our preregistration, its results should be interpreted with caution. Nonetheless, we conclude that R/K judgments, ROC models, and the regression analysis support the DPSD model in Experiment 2, showing that memorability specifically boosts recollection. Whether recollection and familiarity sway on a single continuum of memory strength (Donaldson, 1996; Dunn, 2004, e.g.,), or operate on separate continua (Wixted & Mickes, 2010), or are two qualitatively different processes (Yonelinas, 1994; Eichenbaum et al., 2007) has been an ongoing matter of debate for decades (Yonelinas, 2002; Wixted & Mickes, 2010). Most of the work has shown that both single- and dual-process models generally fit ROC data quite well (Yonelinas & Parks, 2007). From a computational modeling perspective, the DPSD model has one theoretical advantage over single-process accounts: its parameters can disentangle when an item is associated with recollection rather than familiarity. The complexity of natural scenes makes it particularly difficult to understand what aspects of a scene are eventually recollected. Future research could study what images are associated with specific parameter configurations, rather than exclusively relying on fit statistics. Thus, a particularly promising research avenue would be to study ROC curves related to individual images, instead of aggregating data across images.

Our findings have important implications for the application of machine vision algorithms for predicting human memory performance, and for understanding human memory mechanisms. Overall, we show that images differ not only in how accurately people can judge them as old or new (i.e., how memorability has been technically defined), but also in their potential for recollection or familiarity. Importantly, a large portion of this variability is not explained by memorability, i.e., even among the most memorable images that almost every person will accurately recognize as old, some are almost always recognized based on familiarity, others on recollection. This unaccounted variability in the phenomenology of scene memory demonstrates that we have not yet fully understood the nature of intrinsic scene memorability. Given that different neural structures underlie recollective compared to familiar experiences (Eichenbaum et al., 2007), it is likely that the image information that is selectively recollected is differentially processed and represented compared to globally familiar scene information. Machine vision identified scene and object semantics most and least predictive of memorability (Isola et al., 2014). Deep layers in a Convolutional Neural Network (CNNs) identified areas of an image that are most associated with successful memory (Khosla et al., 2015) or single objects that contribute most to the overall memorability score of the entire scene (Dubey, Peterson, Khosla, Yang, & Ghanem, 2015). Thus, a machine can learn the information that comprises successful or unsuccessful memory. As impressive as that is, these algorithms are thus far agnostic regarding the quality of the memory signal it aims to predict. As a case in point, consider a hypothetical scene of a family waiting at a busy airport terminal, accompanied by their two dogs. A machine vision algorithm might predict the scene to be memorable and several image parts and objects will contribute most to that overall memorability: it is an indoor scene, with humans, animals and numerous man-made objects. However, to what extent such information plays a role in human memory and how it is related to the phenomenology of remembering remains elusive. Observers might have a strong sense of familiarity for the overall scene narrative but no particular object or aspect might play a role in the phenomenology of remembering. On the other hand, observers might recollect the image because of associations made to their own autobiographical memories of travels, pets, etc. The CNN feature combinations predictive of memorability likely turn out to be of different relevance for different recognition experiences.

This discrepancy between machine and human intelligence was highlighted in a recent review (Rust & Mehrpour, 2020). More specifically, previous research has shown that neuronal activity for memorable versus non-memorable images is pooled together in the medial temporal lobe (Bainbridge et al., 2017) and in monkey inferotemporal cortex (Jaegle et al., 2019). This finding suggests that memorable scene information might be very close in neuronal representational space, such that object identity is coded by neural spike pattern coding, and memorability is coded by spike magnitude coding (Rust & Mehrpour, 2020). In contrast, Lukavskẏ and Děchtěrenko (2017) showed that memorable scenes are more distant in a multidimensional space representing CNN-based image features. Thus, it is currently unclear how scene representations in a CNN map onto neuronal representations; whatever information allows a computer algorithm to predict a scene’s memorability may be different from the information that people actually remember. Hence, while state-of-the-art machine vision algorithms are powerful tools for predicting scene memorability, their psychological plausibility remains to be determined. Elaborating such algorithms to predict not only recognition accuracy, but also its phenomenology would therefore improve their value as practical tools and as theoretical tools for understanding human memory.

We see a few limitations in this study. First, the model comparison was not optimally tailored to the present models, which are generally difficult to discern based on goodness-of-fit measures only. Bayesian Information Criteria (as well as Akaike Information Criteria) can only capture differences in model complexity when the number of parameters differ between models. However, any given parameter in a model can have strong or small effects on the model’s ability to fit the data. It has been shown that the UVSD model is more complex in its functional form, meaning that, compared to the DPSD, its parameters contribute more to the model’s general ability to fit ROC data (Klauer & Kellen, 2011). For stronger claims about the models’ veracity, experiments should be tailored to specifically study how parameters change under different tasks (Koen et al., 2013) and how model complexity changes fit statistics within participants across multiple experiments (Wixted et al., 2010). Second, we cannot exclude the possibility that some “remember” statements were based on memory strength or strong confidence instead of a recollective experience. Thus, a direct assessment of recollective experience and content with “think aloud” protocols could be a promising avenue for future research.

### Conclusion

Understanding what makes information memorable offers numerous applications to improve the effectiveness of educational materials, marketing strategies, public relations, or pop-culture materials. However, the effect of memorability on the subjective experience of remembering has so far been neglected. We found conclusive evidence that memorability scales with a greater likelihood of episodic recollection but that that there is still considerable variability in the recognition experience: while some memorable images are recognized almost exclusively based on recollection, others are mostly recognized only based on familiarity. This variability is currently not captured by state-of-the-art computer vision algorithms. Which image aspects are differentially associated with the phenomenological experience of recollection? What are the subjective associations that observers make with the information that is more likely to be recollected? Why are certain images highly memorable, but are *consistently* associated with mere familiarity rather than recollection? Our work is an important first step in asking these questions as it emphasizes the importance of considering the phenomenology of remembering both for psychological and computer science research on intrinsic image memorability.

## Appendix

**Table 4:** Number of unique semantic categories per memorability category and indoor/outdoor scene gist, Experiment 1. Note that the absolute number of exemplars per indoor/outdoor scene gist was counterbalanced across memorability categories

Memorability	Scene	Number of unique
Category	Gist	Semantic Categories
High	Indoor	47
High	Outdoor	60
Medium	Indoor	51
Medium	Outdoor	57
Low	Indoor	29
Low	Outdoor	61

**Table 5:** *T* test equivalents of Bayesian analysis framework

Test	*Yold*		*Ynew*		*Yold*_*f*_		*Ynew*_*f*_	
	*t(41)*	*p*	*t(41)*	*p*	*t(40)*	*p*	*t(41)*	*p*
High-Mem vs. Low-Mem	12.20	< .001	14.4	< .001	3.75	< .001	7.35	< .001
High-Mem vs. Zero	4.01	< .001	− .95	.34				

Abbreviations: *Yold* = R/K Scaled Difference, *Ynew* = D/U Scaled Difference. *Yold*_*f*_ = R/K Scaled Difference based on false alarms, *Ynew*_*f*_ = D/U Scaled Difference based on false rejections

**Table 6:** Percentage of unique semantic categories and indoor/outdoor scene gist, Experiment 2. Note that the absolut number of exemplars per indoor/outdoor scene gist was counterbalanced across memorability categories

Semantic Category	Percentage of exemplars in stimuli-set	Indoor/Outdoor Scene Gist
badlands	7%	outdoor
bathroom	16%	indoor
bedroom	9%	indoor
bridge	4%	indoor
conference-room	5%	indoor
dining-room	7%	indoor
conference-room	5%	indoor
dining-room	7%	indoor
golf-course	7%	outdoor
highway	7%	outdoor
kitchen	7%	indoor
lighthouse	4%	outdoor
living-room	7%	indoor
playground	7%	outdoor
skyscraper	8%	outdoor
tower	7%	outdoor

**Table 7:** Frequencies and proportions of phenomenology across confidence levels

Confidence	*Remember*		*Know*		*Detailed-New*		*Unknown*	
	*Freq.*	*Prop.*	*Freq.*	*Prop.*	*Freq.*	*Prop.*	*Freq.*	*Prop.*
Sure	2351	16%	447	3%	1533	10%	703	5%
Medium Sure	346	2%	1006	7%	842	6%	2133	14%
Unsure	131	0.01%	2294	15%	305	0.02%	2984	20%

Abbreviations: *Freq.* = Frequencies, *Prop.* = Proportions

## Footnotes

1 As requested by a reviewer, we analyzed the effect of accuracy feedback on confidence in the subsequent trial. We found a statistically significant difference in confidence judgments after an incorrect vs. correct decision: participants were slightly less confident after an incorrect (*mean confidence* = 1.98) than after a correct decision (*mean confidence* = 2.02) in the previous trial (*X*^2^(1,44) = 6.42, *p* = .01). Note that, by design, accuracy on a given trial was independent of the memorability condition tested on the next trial. Thus, the effect of feedback on confidence does not pose a confound to the effect of memorability on confidence.

2 As requested by a reviewer, we analyzed the effect of accuracy feedback on confidence and on R/K judgments in the subsequent trial. We found no statistically significant difference in confidence judgments or R/K scaled difference scores following an incorrect vs. correct decision

3 The Independence Remember/Know (IRK) procedure proposed by Yonelinas and Jacoby (1995) is a related measure of the probability F that an item is familiar, with F = K/(1-R). It accounts for the fact that, while remember responses are assumed to reflect recollection, know responses do not provide a pure measure of familiarity, but rather the absence of recollection. The scaled difference and the IRK metric are expected to be negatively correlated. Specifically, a condition with a large proportion of K statements will yield a negative scaled difference and a positive IRK measure. This expectation was confirmed by a strong Spearman’s rank correlation between the R/K scaled difference scores and the IRK measures across all images (Spearman’s *ρ* = −.80, *p* < 0.001).

4 This analysis was suggested by an anonymous reviewer who pointed out that model selection only based on fit statistics disadvantages the less flexible DPSD model. However, since this analysis was not preregistered, it should be considered exploratory.

5 One subject did not have a single false alarm for low memorable images and could thus not be included in this test
